# The First 30 Years of the Universal Hepatitis-B Vaccination-Program in Italy: A Health Strategy with a Relevant and Favorable Economic-Profile

**DOI:** 10.3390/ijerph192316365

**Published:** 2022-12-06

**Authors:** Sara Boccalini, Benedetta Bonito, Beatrice Zanella, Davide Liedl, Paolo Bonanni, Angela Bechini

**Affiliations:** 1 Department of Health Sciences, University of Florence, 50134 Florence, Italy; 2 Medical Specialization School of Hygiene and Preventive Medicine, University of Florence, 50134 Florence, Italy

**Keywords:** HBV, immunization, economic evaluation, cost-saving, cost-benefit, ROI, return on investment, BCR, benefit-to-cost ratio

## Abstract

In 1991, Italy was one of the first countries worldwide to introduce a universal hepatitis-B vaccination for children. Since then, epidemiological data have clearly demonstrated the huge clinical benefits of the vaccination. The aim of this study was to update the favorable economic impact of the hepatitis B virus (HBV) vaccination, 30 years after its implementation. A mathematical model was developed to simulate the clinical/economic impact of the universal HBV-vaccination program versus a hypothetical no-vaccination scenario as a posteriori analysis. We assessed the vaccination benefits over a 30-year-immunization-period (1991–2020), and the following period, 2021–2070. Our data showed a big drop in HBV-related diseases (−82% in infections, chronic disease, and hepatocellular-carcinoma cases), and related costs (−67% in the immunization period and −85% in 2021–2070), attributable to vaccination. The return on investment (ROI) and the benefit-to-cost (BCR) ratios are >1 for the first thirty-year-immunization-period, and are predicted to almost triplicate the economic savings in the period 2021–2070, both for the National Health Service (NHS) and from societal perspectives. Our model confirmed that the implementation of universal HBV-vaccination in Italy during the first 30 years continues to be a cost-saving strategy, and more advantageous effects will be further achieved in the future. The HBV-vaccination strategy greatly expresses a huge impact in both the short- and long-term, and from the clinical and economic point-of-views.

## 1. Introduction

Hepatitis B is a liver inflammation caused by the hepatitis B virus (HBV). HBV is a small, enveloped, double-stranded DNA virus (3200 base pair) of the Hepadnaviridae family. When the infection occurs, the virus replicates in the liver cells, producing an excess of the viral-envelope protein (Hepatitis B surface antigen, HBsAg) that circulates in the blood of the host. The HBV transmission-route is both horizontal and vertical. The first occurs by exposure of contaminated blood or body fluids (such as saliva, semen, and vaginal fluids) through the percutaneous and permucosal way. Moreover, transmission may happen in the case of needlestick injury, tattooing or piercing, and the reuse of contaminated needles/syringes or sharp objects in health-care settings, in the community and household contacts, or among drug users. HBV can also spread from mother to child through perinatal transmission [1,2]. In infants and young children, the infection is generally asymptomatic, while approximately 50% of adults develop symptoms [2]. Nevertheless, in a few cases hepatitis B can be fulminant (<1%) [3]. The first-infection age affects the development of the disease: in adults, acute infection is generally self-limited, and less than 5% of cases become chronically infected. Meanwhile, acquiring the infection in the first years of life leads to chronic hepatitis (CHB) in approximately 95% of cases. The main sequelae related to chronic infection are cirrhosis and hepatocellular carcinoma (HCC) [1,2,3].

Different serological markers can describe the development of the acute and the chronic HBV infection: the hepatitis B surface antigen (HBsAg), hepatitis B core antigen (HBcAg), hepatitis B envelope antigen (HBeAg) and their respective antibodies (anti-HBs, anti-HBc and anti-HBe). When the hepatitis B surface antigen persists in serum for more than six months after the infection, the subject is considered chronically infected [4,5]. Moreover, HBeAg can be detected in subjects with acute or chronic HBV infection, and its presence indicates viral replication and thus high viral-levels of HBV DNA and high infectivity. On the other hand, the presence of anti-HBe is generally associated with a decrease of replication activity, although a reversion to HBeAg positivity can occur. Lastly, anti-HBs is considered a marker of protection. These antibodies may be non-detectable in the serum of a chronically infected person [2].

Hepatitis B still remains a global public-health concern. The World Health Organization (WHO) estimates that in 2019, 296 million people were living with chronic HBV infection, with 1.5 million of new infections each year, and that approximately 820,000 deaths occurred due to hepatitis B sequelae, mostly cirrhosis and hepatocellular carcinoma [1]. The distribution of the HBV infection is not homogeneous around the world: in particular, Western Pacific and African regions have the highest prevalence (6.2% and 6.1%, respectively) [6]. In 2019, 30 European Union/European Economic Area (EU/EEA) member states reported 29,996 cases of HBV infection: 6% of them were reported as acute, 48% as chronic, 38% as ‘unknown’, and 7% could not be classified, due to the incompatible data-format provided. The overall rate of acute hepatitis incidence was 0.4 per 100,000 inhabitants. Moreover, the overall notification-rate on chronic-hepatitis-B infections was 4.8 cases per 100,000. The highest rate of acute infections was observed among 35–44-year-olds, and the highest rate of chronic infections among 25–34-year-olds. The overall male-to-female ratio was 1.5:1 [7].

In Italy, according to the latest SEIEVA (*Sistema epidemiologico integrato epatiti virali acute—Acute Viral Hepatis Integrated Epidemiological System*) bulletin, 89 acute-hepatitis-B new cases have been reported in 2021 (0.18 per 100,000 vs. 0.21 per 100,000 in 2020). The most affected age group was the 35–54 year-olds, and males were mostly represented (77.5%). Most of the reported cases occurred in non-vaccinated subjects [8]. The hepatitis B incidence has shown a relevant declining trend from five cases per 100,000 in 1991 to the current level of incidence, following the implementation of universal vaccination for the younger people affected in Italy [9].

As a matter of fact, in 1991 Italy was one of the first countries to introduce the universal anti-hepatitis B vaccination in a double-cohort strategy, for newborns and 12-year-old subjects. Since 2003, anti-hepatitis B vaccination has been kept mandatory just for newborns, in a three-dose schedule (at 3, 5, and 11 months of age) [10].

After the first six years of implementation (1991–1997), a cost-effectiveness evaluation of the Italian HBV-vaccination program was reported by Da Villa et al. This study highlighted for the first time the averted costs following the acute-hepatitis-B case drop, amounting to 2/3 of the expenses for immunization. In particular, the authors specified that the main economic goal would have been reached after 15 years of vaccination (2006), when the National Health Service (NHS) would begin to save money, thanks to the averted treatment of cirrhosis and HCC cases [11]. To confirm this last assumption, more recently, Boccalini et al. performed a new cost-effectiveness study, and remarked on the great economic impact of the universal HBV vaccination 20 years after its introduction in 1991. The results of this economic evaluation showed that the break-even points were just achieved, from both the NHS and societal perspectives. More money-saving should gradually occur from then on [12]. The aim of this study was to update the clinical and economic assessment of the HBV vaccination program in Italy, 30 years after the introduction of the immunization offer, to estimate the savings we obtain with this preventive intervention and the value of the immunization program in the future.

## 2. Materials and Methods

The mathematical model developed in the previous economic analysis by Boccalini et al. [12] was reviewed and updated, for the current aim. This mathematical model simulates the clinical and economic impact since the HBV vaccination strategy started in Italy in 1991, (Microsoft Excel 2010 software—Microsoft Corporation, Redmond, Washington, DC, USA).

We developed a model estimating hepatitis B disease cases and related costs, in a dual vaccination/no vaccination scenario; meanwhile, we calculated the number of hepatitis B vaccine-doses administered to infants and teenagers during 1991–2020, and the total vaccination costs. This study analyzes the burden of the hepatitis B vaccination-program during the past 30-year immunization period (1991–2020) and the following period 2021–2070, considering the latter as a time in which the acute infection acquired in the first 30 years would evolve into more severe outcomes (i.e., chronic hepatitis B, cirrhosis and hepatocellular carcinoma), and thus would need to be treated. We compared the real clinical and economic data to a hypothetical no-vaccination scenario. To do so, we assumed that acute-hepatitis-B incidence in Italy would gradually decrease, thanks to the use of general preventive measures [12]. Specifically, the impact of the vaccination program was performed both from the NHS and the societal perspectives.

The outcomes of the model were estimated considering the different health stages of the HBV disease: symptomatic acute HBV infection (AHB), chronic hepatitis B (CHB), compensated cirrhosis (CC), decompensated cirrhosis (DC), hepatocellular carcinoma (HCC), and the cases of liver transplantation (LT), required in cases of DC and HCC. Our model shows what would be the number of clinical cases with and without the adoption of the vaccination program, (thus, how many cases would be avoided), and also the incidence-reduction rates. Moreover, our model calculates the annual clinical costs (direct and indirect) in a vaccination/no vaccination scenario.

**Figure 1:**
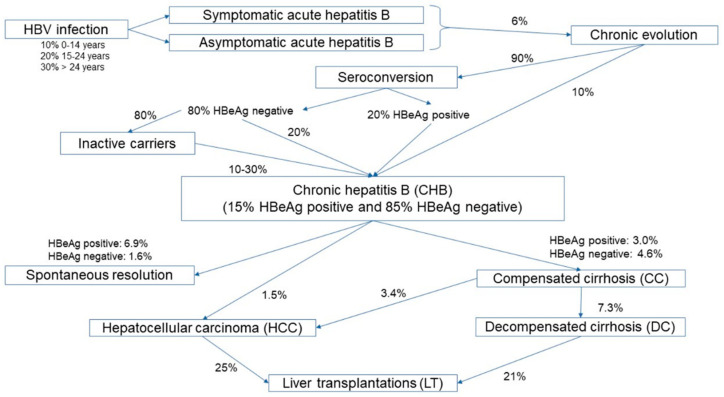
Natural history of HBV infection applied to the mathematical model.

Figure 1 shows the natural history of hepatitis B and the transition rates among the different health stages used in the mathematical model, according to the literature research performed in the previous study [12].

Epidemiological and economic data included in the model have been obtained or updated from the previous study led by Boccalini et al. [12]. In particular, data related to population by age group [13], life expectation [14], acute-hepatitis-B incidence rates [9], the prevalence of HBsAg-positive mothers [15,16,17] anti-HBV vaccine coverage (VC) values [18] and inflation rates [19], were updated.

To calculate the costs associated with the treatment of the diseases, we assumed that cases of AHB were treated only during the first year of diagnosis; on the other hand, costs associated with CHB were applied to all cases, considering the life-long treatment for this disease stage. According to the 5-year survival rate, CC cases were treated as CHB cases for the first 15 years, and then as cirrhosis-affected patients. Similarly, DC patients were treated as CHB for the first 15 years, then as CC for the following 3 years and finally as DC patients. HCC patients were treated as CHB for 20 years (or for 15 years, and then as CC for the following 5 years). They were then treated as HCC patients for 1 year, and subsequently for 4 years as CHB/CC (according to the survival rate), and finally as CHB/CC for 1 year (in line with life expectancy). Patients who require LT are treated simultaneously, both for the transplantation itself and for HCC/DC for 1 year. Subsequently, these patients encounter a post-transplantation follow-up period, calculated as post-transplantation survival rate and life expectancy.

Table 1 reports the time interval between hepatitis B disease stages, the median age of the patients at the time of diagnosis, and the survival rate used in the mathematical model.

Table 2 shows the annual costs of HBV diseases (referring to 1990) and the anti-hepatitis-B vaccination costs (in 1991), reported firstly by Da Villa et al. [11] and updated in line with the annual inflation-rate (provided by the Italian National Institute of Statistical—ISTAT) [19].

Importantly, the major results of the model are expressed as benefit-to-cost ratio (BCR) for the societal perspective, and return-on-investment (ROI) values for the NHS perspective. The BCR or ROI indicates the relationship between the relative costs and benefits; a value of BCR or ROI ≥ 1 represents a favorable outcome, meaning that for each euro invested in the vaccination program the same amount or more would be saved through costs avoided, thanks to the vaccination program.

## 3. Results

### 3.1. Clinical Cases Related to HBV and Related Costs, with and without the Universal HBV Vaccination-Program Implementation

The results of the mathematical model are shown in Table 3. The first thirty years of the HBV vaccination program in Italy (1991–2020) reported a marked reduction rate in the different clinical stages of the disease, compared to a hypothetical no-vaccination scenario. In particular, HBV infections and CHB cases showed a reduction rate of 82%, preventing 193,373 and 6256 cases, respectively. The reduction of the health consequences of CHB was also particularly relevant (−81% for HCC, −79% for LT, −57% for CC and DC). Moreover, the number of AHB cases was halved.

**Figure 2:**
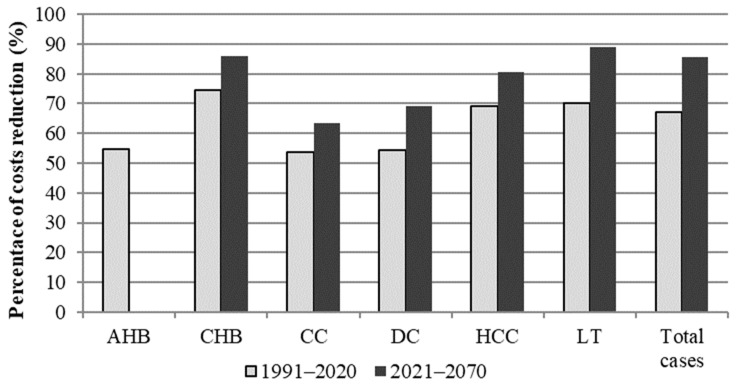
Reduction rate of clinical costs due to implementation of the vaccination program from the NHS perspective (AHB: Acute Hepatitis B; CHB: Chronic Hepatitis B; CC: Compensated Cirrhosis; DC: Decompensated Cirrhosis; HCC: Hepatocellular carcinoma; LT: Liver Transplantation).

Table 4 shows the direct, indirect, and total clinical costs during two periods, 1991–2020 and 2021–2070, and, lastly, the whole period 1991–2070 in the vaccination and no-vaccination scenario. During 2021–2070, only the costs related to the later stages of the HBV infection (CHB, CC, DC, HCC, LT) occurring in the first 1991–2020-period were evaluated. The reduction rate of the direct and indirect costs was comparable in the analyzed periods. Overall, the implementation of the HBV vaccination compared with a no-vaccination scenario resulted in a reduction in clinical costs of 67% up to 2020, 85% during the 2021–2070 period, and 75% in the overall considered-period (1991–2070). Moreover, comparing the reduction rate of clinical costs in the past and in the following period, the highest savings were due to avoided CHB treatment, corresponding to a 75% reduction in 1991–2020, while in the period 2021–2070, costs due to CHB continued to decrease more consistently (−86%). However, it should be noted that the reduction of the clinical costs should be higher for all the sequela of the HBV disease compared to the previous period, especially for liver transplantation (−89%) (Figure 2).

### 3.2. Economic Impact of the Immunization Program

According to our mathematical model, the HBV vaccination of thirty newborn cohorts and twelve 12-year-old cohorts during the first 30 years of implementation (1991–2020) cost EUR 1,263,247,830 from the NHS perspective and EUR 1,484,792,906 from the societal perspective, resulting in net savings of EUR 396,494,926 and EUR 482,577,670 respectively (Table 5). These first thirty years of the HBV immunization-program resulted in an ROI of 1.31 and in a BCR of 1.33, leading to a relevant cost-saving profile both for the NHS and from societal perspectives. ROI and BCR rates are predicted to be higher in the whole period 1991–2070 (2.74 and 2.75, respectively), implying net savings of EUR 2,199,262,351 from the NHS perspective and EUR 2,603,584,165 from the societal perspective.

## 4. Discussion

The aim of this study was to confirm the favorable clinical and economic impact of an extensive universal HBV program in Italy 30 years after its introduction, as reported in the first evaluations [11,12]. As a matter of fact, more than 20 years ago, Da Villa et al. estimated a progressive increase in savings and the achievement of the main economic goal of vaccination starting from 2006, after 15 years of immunization, when money began to be saved in the treatment of cirrhosis and HCC, too [11]. This favorable assumption was confirmed after 20 years of vaccination [12]. The current study demonstrates that 30 years of mandatory anti-hepatitis B vaccination among children in Italy shows a continuous relevant clinical-impact and a cost-saving profile.

Our results related to the relevant clinical impact of immunization on the reduction of HBV disease are in line with the mathematical model developed by Goldstein et al.: the routine pediatric hepatitis-B-vaccination, with 90% of vaccination coverage and the first dose administered at birth, would prevent 84% of global HBV-related deaths [28]. Moreover, with 100% of vaccination coverage and 100% of newborns receiving a birth dose of vaccine, it would be theoretically possible to prevent 95% of all HBV-related deaths [28].

As a matter of fact, in Italy, the hepatitis B epidemiology has considerably changed, with a relevant reduction of incidence rates in these first thirty years of the universal immunization-program, as confirmed by data collected by SEIEVA [9] and many sero-epidemiological studies [29,30]. Moreover, a recent serosurvey highlighted an additional increasing trend of immunity levels acquired by the vaccination in the pediatric and adolescent population [31].

From an economic point of view, our model estimated ROI and BCR values as >1 for the first thirty-year immunization period (1991–2020), which are predicted to almost triplicate the economic savings in the period 1991–2070. This is true both for the NHS and from the societal perspectives. Moreover, the major clinical-costs are now associated with the late stages of the HBV disease, whereas during 1991–2020 they were more related to CHB. This underlines how the extensive vaccination-program has drastically reduced the number of AHB cases, and thus, the number of CHB, CC, DC, HCC and LT cases are now those which are critical to treat. The favorable impact of the anti-hepatitis B vaccination in Italy has also been confirmed by Mele et al. [32], not only for children, but also for certain risk groups, for whom it is recommended.

Our results demonstrated and confirmed how the implementation of the universal hepatitis B vaccination in Italy, which was seen as apparently expensive because of the purchase of many doses of vaccines, instead has produced consistent savings from the NHS and societal point of view in these first 30 years. Moreover, the model predicted that the hepatitis B vaccination will continue to generate savings in the following years, up to 2070. Therefore, the Italian immunization strategy results in value for health, and the current economic benefits could be addressed toward other health priorities, such as prevention activities towards vaccine-preventable diseases and other infectious or chronic diseases, in order to achieve a complete physical, mental and social state of wellbeing.

Savings generated by universal HBV vaccination could be reinvested also, in health promotion and preventive activities related to hepatitis B. For example, greater attention could be paid to protect the high-risk groups. Indeed, some hepatitis B cases occurred in subjects with a higher risk of infection, such as healthcare workers, household contacts of chronic-hepatitis-B carriers, drug users, hemodialysis subjects. and men who have sex with men [32], for whom the HBV vaccination is strongly recommended and free of charge [8,33,34,35]. Moreover, Pinon et al. found a not-always-appropriate surveillance of HBsAg-positive women during pregnancy [36]. Despite Italy providing HBV screening to pregnant women, along with timely immune-prophylaxis and hepatitis-B vaccination at birth to newborns of seropositive women [37], health authorities should consider the possibility of investing more resources also in the screening of pregnant women, in order to prevent HBV vertical-transmission. In addition, in Italy, 21.8% of children are born to at least one parent of foreign nationality [38]. Therefore, the screening of the migrant population would be a good practice to be implemented, especially for people from high-HBV endemic areas [39].

To date, the Italian population aged up to 40 years old has benefited from the universal hepatitis B vaccination, while older age groups of the general population may still be at risk of acquiring the HBV infection, particularly through indirect parenteral-transmission and sexual contact [40]. The latest Italian data highlighted beauty treatments (such as manicures, piercings, or tattoos) as the most frequently reported risk-factors for virus exposure [8,33,34,35,41]. Specific actions should be adopted to reduce hepatitis B cases caused by this way of transmission.

Lastly, it should be taken into account that approximately 5% of fully immunized subjects are non-responders. From a public-health point of view, it could be useful to assess more accurately the impact of screening programs on high-risk groups, such as workers and students in healthcare settings or subjects under chronic disease [42].

Nevertheless, independently by the shown favorable profile, it is crucial to maintain high vaccination-coverage rates in the target immunization-groups in future years, to guarantee the achievement of the hepatitis-B-elimination goal, as desired by the WHO since 2010 [43,44]. In 2016, the WHO released the first global health-sector strategy to end viral hepatitis, which adopted the call for specific actions in the fight against viral hepatitis included in Target n. 3 of the 2030 Agenda for Sustainable Development [45]. Therefore, a broad range of countries have implemented national hepatitis-elimination plans, including Italy [46]. In 2021, the WHO published interim guidance for country validation of viral-hepatitis elimination, focused mainly on hepatitis B and hepatitis C [47]. However, in Italy, after the implementation of the immunization program, HBV-vaccination coverage at 24 months reached a high level (approximately 95%). The HBV vaccination coverage dropped in the period 2014–2016 (reaching 92.9% in 2016) [18,48], and the hepatitis B vaccination was made mandatory to all 0–16-year-olds to attend school [49], in order to increase vaccination coverage. Therefore, the current benefits of the vaccination program could be targeted at maintaining vaccination coverage at high levels, to promote immunization in hesitant subjects, and to contrast vaccination hesitancy.

This study has some limitations. Firstly, as with all pharmacoeconomic analyses, it is based on a mathematical model strongly dependent on the input data used. Therefore, the robustness of our results is related to assumptions. In particular, we used the same data used by the Technical-Scientific and Health Planning Committee of the Ministry of Health. In 1991, this board originally advised and supported the decision to start universal vaccination against HBV infection for all newborns and teenagers in Italy. In order to confirm the first data reported by Da Villa et al. [11], we used the described mathematical-model, although the shift from the monovalent hepatitis-B-vaccine to combined vaccines was not considered. This shift may have reduced the cost of the hepatitis B component. In addition, the effects of co-infections with other viruses, (i.e., HCV, HDV, HIV), or from alcohol and illegal-drug consumption were not included in the analysis. We could not calculate the indirect costs of liver transplantation because of data unavailability; however, we included the medical-assistance costs but not the antiviral-drug-therapy ones (as they are particularly expensive). Therefore, our analysis represents an underestimation of the real costs for chronic-hepatitis management, which are indeed particularly remarkable for the impact of the HBV vaccination. Thus, the real beneficial effects of vaccination could be greater than our estimation. Lastly, this model could be also used to assess the clinical and economic impact of immunization strategies in other settings, (i.e., occupational exposures, hepatitis C infection and treatment, risk groups, etc.).

On the other hand, the main strength of our study is the demonstration of how the universal hepatitis-B-vaccination-program implemented in Italy has generated savings in the short- and long-term which can be reinvested in other public-health activities. In addition, it should be emphasized that this universal preventive-program continued to be favorable in Italy, where the current incidence of HBV was very low, and the specific impact of vaccination is therefore paradoxically reduced by the immunization program itself.

## 5. Conclusions

Despite the availability of a safe and effective vaccine, hepatitis B still represents a major public-health concern worldwide. Nevertheless, countries which implemented a strong immunization program managed to control this infectious disease. Italy was one of the first countries worldwide to introduce a universal HBV-vaccination strategy in 1991, leading to remarkable changes in the hepatitis B epidemiology. Our study confirms that the Italian hepatitis-B immunization-program, in addition to the wide reduction of HBV infections and chronic cases, cut direct and indirect costs in the short- and long-term, and is an effective investment in health value.

## Figures and Tables

**Table 1:** Assumed data used for the model (AHB: Acute Hepatitis B; CHB: Chronic Hepatitis B; CC: Compensated Cirrhosis; DC: Decompensated Cirrhosis; HCC: Hepatocellular Carcinoma; LT: Liver Transplantation).

Stage of the Disease	Time Interval Between Stages	Median Age of Patients	Survival Rate	Reference
CHB	-	31	Equal to life expectancy for the general population	[14,20]
CC	15 years from CHB	46	99.1% at 5 years	[20,21,22,23,24]
			76.8% at 10 years	
			49.4% at 15 years	
			25% at 20 years	
			0% at 25 years	
DC	3 years from CC	49	14% at 5 years	[20]
HCC	44 months from CC	50	78.7% at 1 years	[20,25]
			50.4% at 3 years	
			28.9% at 5 years	
LT	3 months from HCC or DC	-	5% of patients with LT encounter transplant-related death	[26,27]
			72% post-transplant survival rate at 5 years	

**Table 2:** Annual clinical costs of HBV diseases (referring to 1990) and hepatitis-B-vaccination costs (referring to 1991) for Acute Hepatitis B (AHB), Chronic Hepatitis B (CHB), Compensated Cirrhosis (CC) and Hepatocellular Carcinoma (HCC).

Annual Cost of HBV Diseases (1990)	
Annual assistance costs/case	
AHB	9296 EUR
CHB and Cirrhosis (hospital and home assistance)	7230 EUR
HCC	27,889 EUR
Annual social costs/case	
AHB	1916 EUR
CHB and Cirrhosis	1277 EUR
HCC	11,654 EUR
**HBV Vaccination Costs (1991)**	
Direct costs	
Vaccine	6.20 EUR/dose (pediatric dose)
	9.30 EUR/dose (adult dose)
Vaccine preservation	0.09 EUR/dose
Vaccine administration	6.45 EUR/person
Treatment of side effects (1% of vaccine doses administered)	4.93 EUR/case
Immuno-prophylaxis treatment of babies born to HBsAg-positive mothers	14.98 EUR/newborn
Indirect costs	
Lost working-days for vaccination	7.98 EUR/dose
Missed working-days for treatment of side effects (1% of vaccine-doses administered)	21.17 EUR/dose

**Table 3:** Total number of clinical cases related to HBV infection in Italy (AHB: Acute Hepatitis B; CHB: Chronic Hepatitis B; CC: Compensated Cirrhosis; DC: Decompensated Cirrhosis HCC: Hepatocellular carcinoma; LT: Liver Transplantation).

1991–2020	No Vaccination	Vaccination	Prevented Cases	Reduction Rate (%)
HBV infection	237,074	43,701	193,373	82
AHB	61,329	30,931	30,397	50
CHB	7670	1414	6256	82
CC	143	62	81	57
DC	10	4	6	57
HCC	120	23	97	81
LT	32	7	25	79

**Table 4:** Direct, indirect and total clinical-costs (in euros) during the period 1991–2020, 2021–2070 and 1991–2070 in the no-vaccination and vaccination scenario.

1991–2020	Direct Costs (EUR)			Indirect Costs (EUR)			Total Costs (EUR)			Reduction of Direct Costs (%)	Reduction of Indirect Costs (%)	Total Reduction Rate (%)
	No Vaccination	Vaccination	Difference	No Vaccination	Vaccination	Difference	No Vaccination	Vaccination	Difference			
AHB	890,904,717	404,044,092	486,860,625	183,575,866	83,255,530	100,320,337	1,074,480,584	487,299,622	587,180,962	55	55	55
CHB	1,516,037,546	384,847,960	1,131,189,587	267,688,915	67,953,154	199,735,761	1,783,726,462	452,801,113	1,330,925,348	75	75	75
CC	34,501,507	15,965,699	18,535,808	6,091,980	2,819,086	3,272,894	40,593,487	18,784,785	21,808,702	54	54	54
DC	2,000,214	915,589	1,084,625	353,181	161,667	191,514	2,353,395	1,077,256	1,276,138	54	54	54
HCC	23,665,180	7,311,013	16,354,166	4,650,236	1,473,059	3,177,177	28,315,416	8,784,072	19,531,343	69	68	69
LT	8,145,359	2,427,413	5,717,945	1,294,608	364,472	930,137	9,439,967	2,791,885	6,648,082	70	72	70
Total	2,475,254,523	815,511,767	1,659,742,756	463,654,787	156,026,968	307,627,819	2,938,909,310	971,538,735	1,967,370,576	67	66	67
**2021–2070**	**No Vaccination**	**Vaccination**	**Difference**	**No Vaccination**	**Vaccination**	**Difference**	**No Vaccination**	**Vaccination**	**Difference**	**Reduction of Direct Costs (%)**	**Reduction of Indirect Costs (%)**	**Total Reduction Rate (%)**
AHB	0	0	0	0	0	0	0	0	0	0	0	0
CHB	1,984,954,476	280,420,213	1,704,534,263	350,486,247	49,514,198	300,972,050	2,335,440,724	329,934,411	2,005,506,313	86	86	86
CC	13,827,330	5,061,534	8,765,796	2,441,511	893,722	1,547,789	16,268,842	5,955,256	10,313,585	63	63	63
DC	444,275	137,385	306,889	78,446	24,258	54,188	522,721	161,644	361,077	69	69	69
HCC	103,880,973	20,231,671	83,649,303	18,850,137	3,604,567	15,245,570	122,731,110	23,836,238	98,894,873	81	81	81
LT	6,206,322	695,148	5,511,174	446,907	27,434	419,473	6,653,229	722,582	5,930,647	89	94	89
Total	2,109,313,376	306,545,951	1,802,767,425	372,303,249	54,064,179	318,239,070	2,481,616,625	360,610,130	2,121,006,495	85	85	85
**1991–2070**	**No Vaccination**	**Vaccination**	**Difference**	**No Vaccination**	**Vaccination**	**Difference**	**No Vaccination**	**Vaccination**	**Difference**	**Reduction of Direct Costs (%)**	**Reduction of Indirect Costs (%)**	**Total Reduction Rate (%)**
AHB	890,904,717	404,044,092	486,860,625	183,575,866	83,255,530	100,320,337	1,074,480,584	487,299,622	587,180,962	55	55	55
CHB	3,500,992,022	665,268,173	2,835,723,850	618,175,163	117,467,352	500,707,811	4,119,167,185	782,735,524	3,336,431,661	81	81	81
CC	48,328,837	21,027,233	27,301,605	8,533,492	3,712,809	4,820,683	56,862,329	24,740,041	32,122,288	56	56	56
DC	2,444,488	1,052,975	1,391,514	431,627	185,925	245,702	2,876,115	1,238,900	1,637,215	57	57	57
HCC	127,546,153	27,542,684	100,003,469	23,500,373	5,077,626	18,422,747	151,046,526	32,620,310	118,426,216	78	78	78
LT	14,351,681	3,122,561	11,229,120	1,741,515	391,906	1,349,610	16,093,196	3,514,467	12,578,729	78	77	78
Total	4,584,567,899	1,122.057,718	3,462,510,182	835,958,036	210,091,147	625,866,889	5,420,525,935	1,332,148,865	4,088,377,071	76	75	75

**Table 5:** Costs/Savings (in EUR) and ROI/BCR during the first 30 years of the vaccination program (1991–2020) and in the overall period (1991–2070).

Period 1991–2020		
	NHS Perspective	Societal Perspective
Clinical savings (EUR)	1,967,370,576	1,967,370,576
Vaccination costs (EUR)	1,263,247,830	1,484,792,906
Net costs (EUR)	−396,494,926	−482,577,670
ROI/BCR	1.31	1.33
**Overall Period 1991–2070**		
	**NHS Perspective**	**Societal Perspective**
Clinical savings (EUR)	3,462,510,182	4,088,377,071
Vaccination costs (EUR)	1,263,247,830	1,484,792,906
Net costs (EUR)	−2,199,262,351	−2,603,584,165
ROI/BCR	2.74	2.75
